# Evolution of clinically isolated syndrome to pediatric-onset multiple sclerosis and a review of the literature

**DOI:** 10.55730/1300-0144.5434

**Published:** 2022-06-12

**Authors:** İsmail SOLMAZ, İbrahim ÖNCEL

**Affiliations:** 1 Division of Pediatric Neurology, Department of Pediatrics, Faculty of Medicine, Hacettepe University, Ankara, Turkey; 2 Division of Pediatric Neurology, Department of Pediatrics, Faculty of Medicine, University of Health Sciences, Dr Sami Ulus Maternity Child Health and Diseases Training and Research Hospital, Ankara, Turkey

**Keywords:** Pediatric, clinically isolated syndrome, multiple sclerosis, predictive

## Abstract

**Background/aim:**

Clinically isolated syndrome (CIS) may be the first presentation of pediatric onset multiple sclerosis (POMS). We retrospectively evaluated the clinical and laboratory data of pediatric CIS (pCIS) patients who were diagnosed with POMS upon follow-up for any predictive variables. We also reviewed the literature concerning the management of pCIS.

**Materials and methods:**

This single-center study involved patients who had pCIS in childhood that converted to POMS during follow-up between 2011 and 2021. Sixteen patients were included in the study. The data were evaluated retrospectively and analyzed with descriptive statistics.

**Results:**

The majority of the pCIS patients were female (F/M: 10/6, 62/38%), and the first pCIS attack was at 13.3 ± 2.6 years old (mean ± SD). Mean follow-up was 3.1 ± 1.4 years; 6 of the patients relapsed within 1 year and 6 within 2 years. The time from the first pCIS attacks of the patients to the diagnosis of POMS was 15.75 ± 11.07 months. The annualized relapse rate (ARR) was 0.9 ± 0.7. The majority (68%) of the patients had a monosymptomatic onset, optic neuritis (ON) being the most common initial presentation (44%). Cerebrospinal fluid (CSF) oligoclonal bands (OCBs) were found in 9/12 (75%) and the immunoglobulin G index (IgG index) was elevated in 5/11 (45%). An autoimmune disorder was reported in the 1st or 2nd degree relatives of 6 patients: four (25%) MS, one ulcerative colitis, and one Hashimoto’s thyroiditis. Our pCIS patients did not receive any disease-modifying treatment (DMT) for their first attack. When the diagnosis changed to POMS, most (68%) were started on interferons. The Expanded Disability Status Scale (EDSS) increased in one patient during follow-up (EDSS: 3) while in the others it was 0 at the last visit. The literature is reviewed in order to compare results for suggestions regarding the management of pCIS.

**Conclusion:**

The presence of OCBs in the initial episode, MS in the family, and monosymptomatic onset may increase the possibility of developing POMS. Whether DMTs given at the pCIS stage are effective in preventing relapses and disability needs to be evaluated in longitudinal follow-up of large cohorts.

## I. Introduction

Multiple sclerosis (MS) is an inflammatory demyelinating and eventually degenerative disease of the central nervous system (CNS). Pediatric onset multiple sclerosis (POMS) is defined as a disease whose first clinical episodes or symptoms start before 18 years old, and it represents 3%–10% of the total MS cases [1–5]. POMS patients tend to have higher relapse rates [6, 7], higher lesion loads on magnetic resonance imaging (MRI) [8], and more prominent cognitive deficits [9, 10]. The earlier onset and more active course in POMS compared to adult MS may be counterbalanced to some extent by the higher plasticity and regeneration capacity in young individuals [4, 7, 11–15].

The initial demyelinating attack in POMS may present as pediatric clinically isolated syndrome (pCIS) defined as an acute or subacute, clinically monofocal or multifocal CNS event of presumed inflammatory demyelinating nature lasting at least 24 h. Encephalopathy should be absent unless explained by fever or systemic illness, and no past history of CNS demyelinating disease [optic neuritis (ON), transverse myelitis (TM), hemispheric or brain-stem related syndrome] should be reported. The lesions on MRI should not show dissemination in time (DIT) or space (DIS) [16–18]. The diagnostic criteria of the International Pediatric Multiple Sclerosis Study Group (IPMSSG), more suitable for children ≥12 years old, include the presence of cerebrospinal fluid (CSF) oligoclonal bands (OCBs) as evidence for DIT. This facilitates the diagnosis and increases the rate of POMS, concurrently reducing the pCIS group [19].

The treatment of choice during the acute period in pCIS is intravenous methylprednisolone (IVMP) [20] as in other acquired demyelinating syndromes (ADSs), and decisions about long-term treatment differ between centers, being less clear than for adult CIS [20, 21]. Although there is no consensus on maintenance therapy for pCIS, most specialists tend not to start treatment.

Predictive factors for the conversion of pCIS to MS vary between studies and include ON in the first attack, multiple well-defined periventricular or subcortical lesions on MRI, presence of CSF OCBs, disability after the first attack, a short interval between the first two demyelinating episodes, a number of relapses, and progressive onset. In a retrospective report on first ADS in childhood, the presence of OCBs, past infection with Epstein–Barr virus (EBV), periventricular lesions, hypointense lesions on T1, and lesions of the corpus callosum including Dawson fingers were described as predictive [22]. The lower risk of conversion to MS in pCIS cases with negative EBV serology has been supported by others [23]. No consistent correlation was found with sex, age at onset, polysymptomatic vs. monosymptomatic onset, or disease course. In the present study, we aimed to retrospectively evaluate the clinical and laboratory data of patients who were diagnosed with pCIS that then evolved to POMS. We also examined the predictive factors for pCIS progressing to POMS by reviewing the literature.

## II. Materials and methods

Patients who were diagnosed with pCIS at their first ADS episode before 18 years old and diagnosed with POMS during follow-up between July 2011 and July 2021 were included in the study. The diagnoses of pCIS and POMS were made in accordance with the diagnostic criteria of the IPMSSG [17], emphasizing the absence of any previous attacks and the initial presentation not meeting the diagnostic criteria for POMS. The clinical and laboratory data of the patients were evaluated retrospectively using the hospital database. With the patients being under regular follow-up in the clinic, consent could be obtained from the patient and/or the parent for the use of their data for retrospective research. The study was approved by Hacettepe University Ethics Committee (Project number: GO 21/915 Decision number 2021/14–77).

The statistical analyses were performed using a software package (SPSS, IBM SPSS Statistics 24). The data were classified using various parameters. Frequency tables and descriptive statistics were used to interpret the findings.

The literature search was conducted by entering the words “pediatric, multiple sclerosis, clinically isolated syndrome” into search engines such as PubMed, Google Scholar, Embase, and the Cochrane database.

## III. Results

Sixteen patients, 10 female (62%) and 6 male (38%), were included in the study. Age at diagnosis of pCIS was 13.3 ± 2.6 (range 8.2–17.3, median: 13.4) years, age at diagnosis of POMS was 14.6 ± 2.4 (range 9–18, median: 14.2) years, and age at the time of the study was 16.3 ± 2.4 (range 10–19, median: 16.3) years. The time from the first pCIS attacks to the diagnosis of POMS was 15.75 ± 11.07 (range: 2–48, median: 12) months. During follow-up, 9 patients (57%) had a second attack and were diagnosed with POMS. Seven others (43%) who had no further clinical attack fulfilled the MRI criteria due to increasing lesion load on MRI. Therefore, all patients had a 2nd demyelinating attack during mean 3.1±1.4 years’ (range 1–6) follow-up: six patients relapsed in the first year and six others in the second year of follow-up. The annualized relapse rate (ARR) of these 16 patients was 0.9 ± 0.7 (range 0.3–3 median 0.6) during this period.

Eleven patients’ (68%) first episode was monosymptomatic (ON = 5, brainstem = 3, motor = 3) and that of 5 patients (32%) was polysymptomatic (brainstem-cerebellum = 3, brainstem-motor = 1, motor and sensory = 1). A history of autoimmune disease was determined in the 1st or 2nd degree relatives of 6 patients: four (25%) MS, one ulcerative colitis, and one Hashimoto’s thyroiditis. CSF was obtained in 12/16 pCIS patients, for diagnostic purposes during the 1st (n = 4) or 2nd (n = 4) clinical episode, or in the absence of any clinical event (n = 4). Protein and cell counts were normal. OCBs were present in 9/12 (75%) and IgG index was elevated in 5/11 (45%) of the patients tested.

No disease-modifying treatments (DMTs) were given until the establishment of the diagnosis of POMS. Patients who met the diagnostic criteria with a new clinical attack or radiological progression were diagnosed with POMS and DMT was started: 11 (68%) with interferon (IFN), 4 (25%) with teriflunomide, and 1 (7%) with dimethyl fumarate. Three patients showing increased disease activity after receiving IFN had their treatment switched to fingolimod, teriflunomide, or dimethyl fumarate. During the last examination, the Expanded Disability Status Scale (EDSS) scores were 0 in all patients except one, who had a score of 3. Neuropsychiatric testing was not conducted in any patients. All data are summarized in Table.

## IV. Discussion

Our study shows that the majority of patients with pCIS that evolved to POMS afterwards are postpubertal and female and experience about one relapse per year on average. They present monosymptomatically, frequently with ON during the first clinical attack. Autoimmune diseases can be detected in close family members. OCB presence (75%) was more frequent than IgG index elevation (45%) in patients with pCIS evolving to POMS. Disability is rare in the first years. Among ADSs in children, pCIS was the third most common after MS and acute disseminated encephalomyelitis (ADEM) [24]. As in our study, the most common presentation in pCIS is ON in the literature (70%) and the diagnoses may change to ADEM (36%), CIS (24%), MS (19%), or neuromyelitis optica spectrum disorder (NMOSD) (7%) after 28 months’ follow-up [25].

In a multicenter retrospective study on POMS comparing pre-/postpubertal onset, there was female dominance and MS in relatives was found in 6.5% [26]. The distributions of sex and age were similar in our study, but the rate of MS in relatives was higher. This variable is to be compared between larger groups of CIS and POMS. The presence of OCBs in CSF has been associated with high lesion burden on MRI [27], and OCB positivity in CIS cases may have a predictive role [28]. In our pCIS series OCB positivity was 75%. The lumbar puncture was done either at or after the second ADS; only four patients underwent CSF analyses at the initial attack, of which three were positive for OCBs. This finding did not lead to a diagnosis of POMS in these patients because two were before 2017 when OCBs were accepted as marker of DIT and the other did not meet the DIS criteria [19, 29].

The clinical presentation of CIS can be monofocal or multifocal. Derle et al. found onset of monosymptomatic and brainstem/cerebellar nature was common in POMS [30]. Certain studies showed that the presence of brainstem/cerebellar symptoms and ON in the first attack may have a predictive role in the diagnosis of POMS [28, 31, 32]. In our study, brainstem/cerebellar symptoms were more frequent in polysymptomatic pCIS attacks and ON was more frequent in monosymptomatic pCIS attacks. The POMS series from Turkey shows the most common initial clinical presentation was brainstem/cerebellar findings in POMS, with polyfocal onset slightly higher than monofocal (55.4 vs. 44.6%) [26]. According to our results, the first clinical findings of pCIS tend to be monosymptomatic and in the form of ON. More symptoms and signs are expected to develop during the POMS period as the disease is more disseminated compared to pCIS. On the other hand, polysymptomatic CIS might be more prone to evolve into MS; therefore, it would be worthwhile to prospectively follow-up mono- and polysymptomatic ADS cases and compare the rate of developing into POMS.

The following predictive parameters in the conversion of CIS to MS have been studied extensively in adult patients: nonwhite race, female sex, young age, disability after attack, cerebellar syndrome, sphincter dysfunction, cognitive impairment, fatigue, presence of CSF OCBs or serum EBV IgG, MRI lesion load and localization, contrast-enhancing lesions and black holes on MRI, retinal axonal loss, smoking, and decreased vitamin D3 levels [20, 33–37]. In recent years, neurofilament light chain has been reported to be a valuable biomarker predicting progression from CIS to MS in both pediatric and adult studies [38–40].

In recent years, the initiation of DMT in CIS tends to be favored because of reports on EDSS remaining stable under treatment. The rate of CIS converting to MS can be as high as 85% in adults [41] and interferon, glatiramer acetate, teriflunomide, and cladribine might have a beneficial effect on conversion rates to MS [20]. Although not directly on the maintenance treatment regimen in pCIS, certain large-participant DMT studies included pCIS cases [42]; confirmation by direct clinical trials in pCIS is needed [43, 44]. Over 2.2–7.6 years’ follow-up, the probability of pCIS turning into clinically definite POMS can vary between 15% and 62% [45–50]. However, no DMT regimen is approved by health authorities for preventing pCIS developing into POMS [51, 52]. In the absence of evidence-based data and guidelines on long-term treatment in pCIS, opinions and practice vary on this issue [53].

Cognition is another function to be monitored in POMS. Processing speed can be impaired in 23.4% of POMS patients and in 16.4% of pCIS patients [54]. If DMTs are shown to prevent cognitive impairment when started at the stage of pCIS, early prescription of DMT may be justified [54, 55]. For this reason, baseline assessment and follow-up of cognitive functions are recommended in POMS, with early initiation of DMT whenever cognition appears affected, especially in patients with pCIS with a high risk of converting into POMS. According to the National Multiple Sclerosis Society recommendations, although there is no definite consensus regarding the advantages or disadvantages of using DMT for cognitive impairment, in long-term follow-up all of the approved DMTs have been shown to reduce the number and severity of MS attacks and attenuate the signs of brain damage seen on MRI, they may have beneficial effects on cognitive function.^1^ Cognitive impairment is not listed as an indication for starting DMT in POMS in many health insurance systems. This issue needs to be addressed by health authorities.

The largest studies of pCIS are of multicenter, retrospective nature; follow-up of 770 pCIS patients for at least 10 years revealed significantly higher incidence of worsening EDSS in patients with initially multifocal, isolated spinal cord, or ON attacks in comparison to those with an initial supratentorial or brainstem syndrome. Early DMT exposure prevented the second attack and worsening of disability. Authors described these as novel findings clearly demonstrating the importance of early treatment in pCIS and POMS [56] as already reported for adult CIS [57, 58]. Another retrospective observational study confirmed abnormal cranial MRI, presence of OCB, and age as independent predictors of conversion to POMS in a series of children with isolated ON [59]. In our study, OCB positivity, ON at monofocal onset, and brainstem or cerebellar system involvement in polyfocal onset appeared to have a predictive role in the transition from pCIS to POMS, and the fact that the patients were on DMT in the early phase of POMS may have prevented worsening of their EDSS. The role of DMT, especially newer DMTs, in preventing disease activity in pCIS has been suggested [42]. However, healthcare regulations vary in different countries and insurance coverage is an important factor directing the treatment of POMS and particularly pCIS [60].

Although our study is limited by its retrospective nature, the small size of the series, the relatively short follow-up period, the lack of neuropsychiatric tests to evaluate cognitive status, and the absence of detailed MRI and laboratory analyses, it allows a look at the early stages of pCIS evolving into POMS, suggesting that OCB positivity, family history, monosymptomatic presentation, and ON are associated with POMS, and will serve as a basis for further studies of natural course and management.

## Figures and Tables

**Table:** Clinical data of pediatric clinically isolated syndrome patients.

Cohort n = 16	10F (62%) / 6M (38%)
Age at pCIS	13.3 ± 2.6 years (range 8.2–17.3, median: 13.4)
Age at POMS	14.6 ± 2.4 (range 9–18, median: 14.2)
Current age	16.3 ± 2.4 (range 10–19, median: 16.3)
ARR	0.9 ± 0.7 (range 0.3–3 median 0.6)
Symptomatology	Monosymptomatic n = 11 (68%). ON = 5 (44%), brainstem = 3 (28%), motor = 3 (28%) Polysymptomatic n = 5 (32%). Brainstem-cerebellum = 3 (60%), brainstem-motor = 1 (20%), motor and sensory = 1 (20%)
First DMTs	IFN-β = 11 (68%), teriflunomide = 4 (25%), dimethyl fumarate = 1 (7%)
Treatment switch	IFN-β to Fingolimod, Dimethyl fumarate, Teriflunomide, one each
OCB n = 12	9/12 positive (75%)
IgG index n = 11	5/11 had elevation (45%)
EDSS score	3 (n = 1), 0 (n = 15)

**F:** Female, **M:** Male, **pCIS:** Pediatric Clinically Isolated Syndrome, **POMS:** Pediatric Onset Multiple Sclerosis, **ARR:** Annualized Relapsing Rate, **ON:** Optic Neuritis, **DMT:** Disease Modifying Treatment, **IFN-β:** Interferon Beta, **OCB:** Oligoclonal band, **IgG:** Immunoglobulin, **EDSS:** Expanded Disability Status Scale.

## Footnotes

1 National Multiple Sclerosis Society, Managing Cognitive Problems in MS [2016]. Website https://www.nationalmssociety.org/NationalMSSociety/media/MSNationalFiles/Brochures/Brochure-Managing-Cognitive-Problems_1.pdf [Accessed 02 June 2022].
